# The Association between Work-Related Rumination and Heart Rate Variability: A Field Study

**DOI:** 10.3389/fnhum.2017.00027

**Published:** 2017-01-31

**Authors:** Mark Cropley, David Plans, Davide Morelli, Stefan Sütterlin, Ilke Inceoglu, Geoff Thomas, Chris Chu

**Affiliations:** ^1^Department of Psychology, University of SurreyGuildford, UK; ^2^Center for Digital Economy, University of SurreyGuildford, UK; ^3^BioBeats Group LTDLondon, UK; ^4^Department of Computer Science, University of PisaPisa, Italy; ^5^Department of Psychology, Lillehammer University CollegeLillehammer, Norway; ^6^Department of Neurobiological Medicine, Oslo University Hospital RikshospitaletOslo, Norway; ^7^Surrey Business School, University of SurreyGuildford, UK

**Keywords:** work-related rumination, heart rate variability, unwinding from work, recovery

## Abstract

The objective of this study was to examine the association between perseverative cognition in the form of work-related rumination, and heart rate variability (HRV). We tested the hypothesis that high ruminators would show lower vagally mediated HRV relative to low ruminators during their leisure time. Individuals were classified as being low (*n* = 17) or high ruminators (*n* = 19), using the affective scale on the work-related rumination measure. HRV was assessed using a wrist sensor band (Microsoft Band 2). HRV was sampled between 8 pm and 10 pm over three workday evenings (Monday to Wednesday) while individuals carried out their normal evening routines. Compared to the low ruminators, high affective ruminators demonstrated lower HRV in the form of root mean square successive differences (RMSSDs), relative to the low ruminators, indicating lower parasympathetic activity. There was no significant difference in heart rate, or activity levels between the two groups during the recording periods. The current findings of this study may have implications for the design and delivery of interventions to help individuals unwind post work and to manage stress more effectively. Limitations and implications for future research are discussed.

## Introduction

Perseverative cognition has been conceptualized as “the repeated or chronic activation of the cognitive representation of stress-related content” (Brosschot et al., 2005). Consistently, studies have shown that it may not be the actual stressors themselves that cause health issues but that it is the continued mental representation of the stressor/s—in the absence of the actual stressor—that is the main contributor. Prolonged perseverative cognition has been associated with a range of negative physical (Brosschot et al., 2006, 2010; Verkuil et al., 2010; Ottaviani et al., 2016), and psychological conditions (Garnefski and Kraaij, 2006; Aldao et al., 2010).

The fundamental principle of the perseverative cognition hypothesis is that disease risk is increased when individuals continue to experience unwanted mental representation of a stressful situation, which in turn elicits prolonged physiological arousal. There are many different possible stressors (e.g., work, home, private); in the present article, we focus solely on the role of mental presentations associated with work, in the form of work-related rumination. Work-related rumination has been defined as a thought or thoughts directed to issues relating to work, that is/are repetitive in nature (Cropley and Zijlstra, 2011). For example, a worker may ruminate about an important project deadline, an unfinished task, stress over a future meeting, or they may perseverate about something negative that was said to them by their line manager or colleague at work (Cropley and Millward, 2009; Syrek et al., 2016).

Workers need to psychologically detach and unwind from the demands of work during their leisure time to replenish lost resources that were expended at work (Meijman et al., 1998). Delayed psychological recovery in terms of repetitive or ruminative thinking about work has been associated with a range of health complaints (Pravettoni et al., 2007; Gustafsson et al., 2008; Rydstedt et al., 2009; Sonnentag et al., 2010; Querstret and Cropley, 2012; Cropley et al., 2013), but importantly, and of relevance for the present study, failure to unwind from work has been associated with increased cardiovascular disease risk (Suadicani et al., 1993; van Amelsvoort et al., 2003; Kivimäki et al., 2006). For example, a prospective study by Suadicani et al. (1993) found that men who reported an inability to relax after work had an approximately threefold increased risk of Ischemeic heart disease. Another study of 788 industrial workers found that incomplete recovery at weekends was predictive of cardiovascular death (Kivimäki et al., 2006). What was interesting about this study was that the initial cohort was free from cardiovascular disease and that the findings remained significant after controlling for conventional risk factors, including age, sex, cholesterol, systolic pressure, body mass index, smoking, physical inactivity, fatigue and job stress.

The exact mechanism underlying the association between work-related rumination and cardiovascular disease risk is not clear; however, two distinct pathways may be involved: the behavioral and the physiological. The behavioral pathway can be influenced by many factors such as leading a sedentary lifestyle, drinking alcohol to excess, smoking and moderating eating habits, and there is some evidence to suggest that high work-related ruminators consume more foods that contain saturated fats and sugars than low ruminators (Cropley et al., 2012). Regarding the physiological pathway, although not fully understood, two involuntary branches of the autonomic nervous system appear to be closely involved in the progression from stress to disease: the sympathetic and the parasympathetic nervous systems. When the body is under threat or stressed, sympathetic activity (or parasympathetic withdrawal) mobilizes the organism for action by initiating physiological arousal, such as increasing blood pressure, heart-rate, catecholamine and corticosteroid secretion. In the absence of threat or perceived stress, the parasympathetic system counteracts the effects of sympathetic activity and restores homeostasis. These two mechanisms serve to protect the organism in the short-term but can have damaging effects if stress is prolonged such as when people perseverate about work matters.

There are various ways to assess autonomic nervous system activity and many of these are quite intrusive. However, one non-invasive marker that is the subject of this article is heart rate variability (HRV). HRV is considered as an objective, discreet measure of vagally mediated cardiac regulation and a predictor of cardiac disease risk (Tsuji et al., 1996; Hansson and Jönsson, 2006). As a proxy for prefrontally modulated and vagally mediated cortico-cardiac interaction, HRV is thought to be a marker for emotional regulation, responding when individuals are under mental stress (Appelhans and Luecken, 2006; Heponiemi et al., 2006; Thayer and Lane, 2009; Geisler et al., 2010; Forkmann et al., 2016). Vagally mediated HRV can therefore be construed as an indicator of how well people regulate their emotions (Koval et al., 2013).

The purpose of the present study was to examine the effects of perseverative cognition in the form of work-related rumination on HRV. There are a number of studies that have examined rumination and HRV (Ottaviani et al., 2016), but to our knowledge, and following a review of the literature, only one study has examined the effects of work-related rumination on HRV (Vahle-Hinz et al., 2014). Vahle-Hinz et al. (2014) assessed the effects of work stress, work-related rumination, sleep and nocturnal HRV on a work day and over the weekend. No significant associations were observed between work-related rumination and nocturnal HRV on a workday, although work-related rumination measured on Saturday evening was related to nocturnal HRV. However, it was a positive predictor, suggesting greater HRV with high rumination. As the authors state, this finding is quite perplexing, and although they present a rational argument for why this is, they also offer some compelling limitations which could have attributed to these results. In their study, rumination was only measured by one item, “Today I had to think about work-related problems at home” and as the authors point out this only assessed whether workers thought about work in the evening or not, whereas HRV seems to be particularly sensitive to emotional regulation. It was their suggestion that future research should include measures of rumination that explicitly assess affective reactions related to work.

To this end, the present study investigated the association between work-related rumination and HRV over three evenings. Following Vahle-Hinz et al. (2014) suggestion, we chose a measure of affective rumination by Cropley et al. (2012). This measure specifically assesses affective reactions related to thinking about work. It was predicted *that individuals reporting high affective work-related rumination would demonstrate lower HRV during a workday evening relative to low ruminators*. In order to test this hypothesis, we captured continuous data from a wrist band photoplethysmography (PPG) sensor, first passing the sensor data through a paired smartphone and then uploading to a cloud instance, where further analysis could be performed. To our knowledge, this is the first study to use wrist band PPG in order to capture HRV data in a field study.

## Materials and Methods

### Sample and Participants

The sample was recruited from a financial sector organization (BNP Paribas) in collaboration with AXA/PPP, who made it possible for us to interact directly with the employee population. Participants were drawn from a larger cohort of full-time working adults. One hundred and ninety-five individuals (*F* = 29.8%, *M* = 70.2%) with an age range of 20–62 years (*M* = 38.69, SD = 9.44) completed the affective subscale of the work-related rumination questionnaire and low (*n* = 55) and high (*n* = 49) ruminators were identified using quartile splits. As both HRV and tendencies to ruminate have been known to be affected by age (Britton et al., 2007; Sütterlin et al., 2012; Vahle-Hinz et al., 2014), we restricted the upper age eligibility to 45 years. Due to missing data (due to removal of the band from the wrist, signal loss and movement artifacts), the final sample consisted of 19 high ruminators (mean age 34.3 years, SD = 6.7 years, 31.6% females) and 17 low ruminators (mean age 33.3 years, SD = 6.55 years, 17.6% females).

#### Work Related Rumination

The affective rumination subscale of the work related rumination questionnaire was used to assess people’s perseverative cognitions about work (Cropley et al., 2012). Items are responded to on a 5-point Likert scale ranging from 1 = “Very seldom/never, seldom, sometimes, often to 5 = Very often/always”, e.g., “Are you troubled by work-related issues when not at work?”; This measure has been used in a number of previous studies (Querstret and Cropley, 2012; Querstret et al., 2016), and has shown good reliability and validity (Querstret and Cropley, 2012; Syrek et al., 2016).

### Heart Rate Variability Assessment

The Microsoft Band v2 was selected to capture interbeat intervals because it exposes peak-to-peak (PP) intervals (the duration of every detected heartbeat) through its developer SDK, while most wearables only provide average heart rate, and from which we can derive HRV. Whilst deriving HRV from PPG data is quite novel, there is a precedent for ECG/PPG correlation when looking at HRV data (Selvaraj et al., 2008). Moreover, the device can be programmed to turn on and off the sensors without any explicit interaction with the user, i.e., accelerometer and heart rate sensors can be turned on and off while the user is sleeping, which makes it possible to acquire and analyze data without the potential bias introduced by interaction, which can in itself be a form of intervention. We created an app for iOS, that pairs with the Microsoft Band v2, and periodically collects the data from the sensors, and uploads the collected data to our cloud, for analysis. We implemented an algorithmic policy to balance the amount of captured data and battery consumption to better preserve battery life. The heart rate was captured for three consecutive minutes (in order to acquire a continuous stream of PP data long enough to ensure a minimum level of validity in HRV analysis), then turned off for 3 min (to avoid depleting the battery unnecessarily). Accelerometry data was measured along HRV to assess movement. The accelerometer was periodically turned on for 15 s, then turned off for 45 s. The accelerometer captures data from a triaxial accelerometer and a triaxial gyroscope, at a sampling frequency of 60 Hz. To keep the communication from the smartphone to the cloud to a minimum, we uploaded a small cluster of statistical features of the accelerometer data, instead of all the raw data. The uploaded features provided sufficient information to allow us to discriminate between user activities (still, walking, running, automotive, other). The Microsoft Band’s PPG sensor is heavily affected by motion artifacts. To account for this, we filtered the collected heart rate data retaining only the data collected when the accelerometer reported “stationary” activity, discarding the data collected while the accelerometer reported “walking”, “running”, “automotive” or “other”. This filtering step was necessary to ensure that the collected data did not contain noise that would have corrupted the subsequent HRV analysis.

### Procedure

The Ethics Committee of the University of Surrey granted a favorable ethical opinion for the research. Information about the study was circulated via the intranet. Interested participants within BNPP were provided with further written information, and once consented, completed the affective work-related rumination measure online. Individuals who chose to participate were each sent a wrist band that was paired to their company-issued smartphone, with an instruction sheet on how to use them together. They were also provided with a help line to a research assistant who could guide them through the set-up procedure if needed. No additional considerations are appropriate as participants were otherwise healthy adults in full time work. Individuals were asked to wear the wristband continually (apart from when bathing). As research shows that people ruminate about work issues more at the beginning of the week compared to the rest of the week (Cropley, 2015) the analysis presented here represents data collected over three weekday evenings (Monday–Wednesday). Theoretically, HRV can be modulated by voluntary actions which could bias the results for example, by practicing breathing exercises individuals can deliberately reduce their heart rate (and improve their HRV); individuals were therefore asked to behave as they normally would during their leisure time, and no self-management intervention or visibility over the data collected was offered during this data collection period.

### Heart Rate Variability Analysis

All data were screened for measurement artifacts using ARTiiFACT software (Kaufmann et al., 2011). Artifacts were identified using the algorithm developed by Berntson et al. (1990) by identification of a distribution-based threshold value calculated for each individual. Flagged beat intervals were visually checked and if confirmed as artifacts deleted and substituted by means of cubic spline interpolation of neighboring intervals. The time-domain measure root mean square successive difference (RMSSD), which is considered to indicate vagally mediated HRV, was calculated for each person. Measurements and analyses followed established guidelines (Task Force of the European Society of Cardiology and the North American Society of Pacing and Electrophysiology, 1996).

Previous work has demonstrated that most workers do not instantaneously switch off as soon as they stop working, but gradually cognitively disengage and relax over the course of the evening (Cropley and Millward Purvis, 2003; Cropley et al., 2006). To ensure that individuals were given adequate time to disengage from work, HRV measurements were sampled between an 8 pm and 10 pm window (10 pm was chosen as the upper time period to minimize the possibility of individuals being asleep when the HRV data was sampled). A continuous 3-min stream of PP data was captured for analysis for each individual as close to 8 pm as possible, alongside concomitant accelerometer data. Prior to analysis, the data was screened for normality and RMSSD was found to be normally distributed. Data analysis was conducted using between groups ANOVAs.

## Results

RMSSD was significantly lower in the high ruminators (*M* = 139.0; SD = 45.819) relative to the low ruminators (*M* = 178.1; SD = 63.501), *F* = 4.556, *p* < 0.02, *η*^2^ = 0.18. There was no significant difference between the groups with respect to physical activity (0.993, SD = 0.276, vs. 0.992, SD = 0.022), nor was there a significant difference in the time during the evening when HRV was sampled (20.21, SD = 0.36, vs. 20.24, SD = 0.30). Finally, there was no significant difference in heart rate between the two groups (*M* = 78.539; SD = 11.647, vs. 82.953; SD = 12.592), *F* = 1.194, ns, *η*^2^ = 0.03. When the analysis was repeated controlling for age and gender, the overall pattern of the results was unchanged.

## Discussion

Research has demonstrated that irregularity of heartbeat intervals is an important marker of health (Kemp and Quintana, 2013). This study adds to this literature as rumination was associated with reduced HRV, which is a known indicator of cardiovascular disease risk (Tsuji et al., 1996). As predicted, individuals reporting high affective rumination were found to demonstrate lower HRV compared to low ruminators. As vagally mediated HRV is recognized as an indicator of how well people regulate their emotions, this suggests that high affective ruminators are less able to regulate their emotional thoughts about work after work, reflecting lower parasympathetic activity during the working week.

To our knowledge, this was the first study to pair wearable sensors with smartphones with a particular focus on examining the association between work-related rumination and HRV. There was noise in the data, which is to be expected when collecting the data in the field, and this led to a loss in sample size and power. Also, data capture was limited to avoid depleting the battery unnecessarily. As it is theoretically possible for people to modulate their HRV, it was deemed important for participants to behave normally during their evenings and not to deliberately practice breathing exercises, as this could have potentially biased the findings. Given that there is inherent and unavoidable loss of data introduced by movement in PPG-acquired HR signal, it may be advisable for future studies to design data collection methods that incorporate periods of passivity, where continuous measures can be taken while participants are completely sedentary, or to use larger samples in order to compensate for the loss of power. In field work, it is impossible to have the same level of control as in the laboratory, and a compromise will always be needed.

Although comparable in size to other HRV studies (Steinmetz et al., 2016; Wojniusz et al., 2016) the current findings were based on a relatively small sample size, and in addition the HRV data was high for both groups, therefore some caution needs to be exercised in the interpretation of the results until the findings are replicated in larger samples. In addition, we did not control for potential confounding factors such as smoking, exercise, use of medication or somatic and psychological illness. Future field studies should include these potential confounders of current health status and health-related behaviors.

As the study had a quasi-experimental design, causality cannot be inferred from the data; nonetheless, the study may have clinical implications. HRV biofeedback has seen a resurgence in recent years, and biofeedback interventions have been shown to be effective in individuals with emotional disturbance (Wheat and Larkin, 2010). Interventions that include mindfulness breathing exercises have also been shown to reduce work-related rumination (Hülsheger et al., 2014; Querstret et al., 2016). It is now possible for mobile health interventions to incorporate biofeedback breathing exercises to help people regulate their emotions during acute periods of stress. mHealth interventions providing this kind of physiological monitoring can offer increased fidelity, portability and functionality over traditional home-based biofeedback monitors (Luxton et al., 2011), and an increasing number of studies show evidence supporting the greater use of technological innovations in psychological care (Hollis et al., 2015). Early precedents for vagal modulation as a self-management mechanism for stress (Benson et al., 1974) have been reinforced by more recent work in neurocardiac training for hypertension to enhance vagal heart control (Nolan et al., 2005). Future research might examine whether such interventions could be incorporated alongside HRV data collection methods to provide instant feedback to users, improving their efficacy, and potentially incorporating findings from computational intelligence research in affect modeling for gameplay (Plans and Morelli, 2012) to have intervention content adapt in real-time to user affect.

Ruminating about work has been associated with negative health and increased risk of disease. To our knowledge, this is the first study to demonstrate an association between a trait marker of work-related rumination and a short-term HRV parameter. This was also the first study to pair smartphone technology with a wearable wristband to record HRV during daily life. The current study is not void of limitations; nonetheless, it does provide further support for the role of HRV as a proxy indicator for how well people regulate their emotions outside of work, and also shows that failure to unwind from work is a possible risk factor for the development of cardiovascular disease.

## Author Contributions

MC and DP made equal contributions to this work. MC, DP and DM made substantial contributions to the conception and design of the work. MC, DP, DM and SS drafted the work and revised it critically for important intellectual content; have provided approval of the version to be published and agree to be accountable for all aspects of the work in ensuring that questions related to the accuracy or integrity of any part of the work are appropriately investigated and resolved. II, GT and CC made important contributions to the research design contextualization of psychometric data in the workforce for the trial.

## Funding

The University of Surrey’s Impact Acceleration Account (IAA), funded by the Engineering and Physical Sciences Research Council, provided funding for the purchase of an initial set of wearable devices used in early prototyping for this work, as well as research assistant support. AXA/PPP and BNPP provided funding for the wearable devices used during this study, the early part of which generated the data used in this article.

## Conflict of Interest Statement

Some of the authors of this article (DP and DM) are employees of a commercial company, BioBeats Group Ltd., which holds a patent for technologies used in the present research (US Pat. No. 9,330,680) pertaining to biometric data gathering from smartphones and its use in biofeedback applications. The other authors declare that the research was conducted in the absence of any commercial or financial relationships that could be construed as a potential conflict of interest.
